# Modulation of cell wall synthesis and susceptibility to vancomycin by the two-component system AirSR in *Staphylococcus aureus* NCTC8325

**DOI:** 10.1186/1471-2180-13-286

**Published:** 2013-12-10

**Authors:** Haipeng Sun, Yifan Yang, Ting Xue, Baolin Sun

**Affiliations:** 1 Department of Microbiology and Immunology, School of Life Sciences, University of Science and Technology of China, Huangshan Road, Hefei, Anhui 230027, China

**Keywords:** Vancomycin, Two-component system, Cell wall, Autolysis

## Abstract

**Background:**

Vancomycin has been the medication of last resort to cure infections caused by *Staphylococcus aureus* since the increase in the prevalence of methicillin-resistant *Staphylococcus aureus* (MRSA). Some strains have developed vancomycin-intermediate resistance, which is generally associated with altered expression of or mutations in some part of the two-component system (TCS), such as GraSR, VraSR, and WalKR.

**Results:**

We deleted the AirSR TCS in *S. aureus* NCTC8325 and compared the resultant transcript levels with those of its parent strain using microarray analysis. The results indicated that more than 20 genes that are related to cell wall metabolism were down-regulated in the *airSR* mutant. The *airSR* mutant exhibited reduced autolysis rates and reduced viability in the presence of vancomycin. Real-time reverse transcription PCR and DNA mobility shift assays verified that AirR can directly bind to and regulate genes that function in cell wall metabolism (*cap*, *pbp1,* and *ddl*) and autolysis (*lytM*).

**Conclusions:**

AirSR acts as a positive regulator in cell wall biosynthesis and turnover in *Staphylococcus aureus* NCTC8325.

## Background

*Staphylococcus aureus* is a major human pathogen that can cause a number of types of infections and inflammations, ranging from superficial skin infections to life-threatening toxic shock syndrome, septicemia, osteomyelitis, and endocarditis [1]. *S. aureus* has developed many defense mechanisms to protect itself from the human immune system and antibiotic treatment. Methicillin-resistant *Staphylococcus aureus* (MRSA) has been spread worldwide, rendering the entire β-lactam class of antibiotics ineffective [2]. So far, vancomycin has been the most reliable therapeutic agent against infections caused by MRSA. Vancomycin binds to D-alanyl-D-alanine residues of the murein monomer to interfere the synthesis of bacterial cell wall [3]. The cell wall is very important for *S. aureus* to maintain an osmotic gradient, and a thickened cell wall is often related to increased resistance to vancomycin [3]. In addition to the cell wall, *S. aureus* is encapsulated by capsular polysaccharides, which can protect cells from phagocytosis [4].

Two-component systems (TCSs) act as a basic stimulus–response to allow organisms to sense and respond to changes in many different environmental conditions. Typical TCSs have two components, a histidine protein kinase and a response regulator. The kinase senses the environmental stimuli, autophosphorylates at a histidine residue, and transfers the phosphoryl to an aspartate residue in the response regulator. Then the regulator is active to regulate downstream genes [5]. Bioinformatics analysis indicates that *S. aureus* harbors 16 conservative TCSs. In many cases, virulence gene expression is controlled by TCSs such as the well-studied AgrAC [6,7] and SaeSR [8]. In addition to virulence control, the TCSs are involved in the regulation of biofilm formation [9], autolysis [10], heme toxin resistance [11], cell wall synthesis [12,13], capsular polysaccharide synthesis [14], and antibiotic resistance [15-17]. In *S. aureus*, WalKR is a well-known TCS for its role in controlling cell wall metabolism and cell survival [12]. Recently, WalKR has been reported to be involved in vancomycin resistance [18]. By introducing a point mutation of WalK, *S. aureus* exhibited reduced susceptibility to vancomycin [19]. The TCS VraSR, can positively modulate cell wall biosynthesis and increase resistance to vancomycin [13,15]. Another TCS, GraSR, can modulate vancomycin resistance partly by regulating an adjacent ABC transporter, VraFG [16].

Although most TCSs in *S. aureus* have been well studied, the function of a few TCSs remains elusive or only partially explained. AirSR (YhcSR) was first reported to be an essential TCS [20] and was involved in the regulation of the nitrate respiratory pathway [21]. Subsequently, AirSR was described as an oxygen sensing and redox-signaling regulator [22]. A recent study demonstrated that AirSR can regulate the *lac* and *opuCABCD* operons [23]. It appears that more work is needed to address the function of this TCS. In this study, we deleted *airSR* in *S. aureus* NCTC8325 and observed that approximately 30 cell wall metabolism-associated genes were down-regulated in the *airSR* mutant in our microarray result. After further investigation of cell wall-related phenotypes, we found that inactivation of *airSR* led to reduced autolysis rates and reduced viability in sub-inhibitory concentrations of vancomycin. Real-time reverse-transcription (RT) PCR verified the down-regulation of several cell wall-related genes and the autolysin LytM. Electrophoretic mobility shift assays indicated that AirR can directly bind to the promoter regions of *cap*, *ddl*, *pbp1*, and *lytM*, indicating that *airSR* is directly involved in cell wall biosynthesis and turnover processes and, subsequently, vancomycin susceptibility.

## Methods

### Bacterial strains, plasmids, and growth conditions

The bacterial strains and plasmids used in this study are listed in Table 1. *Escherichia coli* DH5α and BL21 (DE3) were grown in lysogeny broth (LB) medium (BD, Franklin Lakes, NJ, USA) with appropriate antibiotics (100 μg/ml ampicillin sodium salt or 50 μg/ml kanamycin sulfate). *S. aureus* and its derivative strains were grown in tryptic soy broth (TSB) medium (BD) with erythromycin (2.5 μg/ml) or chloramphenicol (15 μg/ml) when necessary.

**Table 1:** Strains and plasmids used in this study

**Strain or plasmid**	**Relevant genotype**	**Reference or source**
Strains		
NCTC8325	Wild-type	NARSA^a^
RN4220	8325-4 r- initial recipient for modification of plasmids which are introduced into *S. aureus* from *E. coli*	NARSA
ΔairSR	8325 *airSR::ermB*	This study
CairSR	8325 *airSR::ermB* pLIairSR	This study
DH5α	Clone host strain, *supE44 ΔlacU169* (*φ80dlac*ZΔM15) *hsdR17 recA1 endA1gyrA96 thi-1 relA1*	TransGen
BL21 (DE3)	Express strain, F^-^*ompT hsdS*_*B*_ (r_B_^-^ m_B_^-^) *gal dcm*(DE3)	TransGen
Plasmids		
pEasy-blunt simple	Clone vector, Kan^r^ Ap^r b^	TransGen
pET28a(+)	Expression vector with a hexahistidine tag, Kan^r^	Novagen
pEairR	pET28a(+) with the *airR* coding sequence, Kan^r^	This study
pEairS	pET28a(+) with the *airS* coding sequence, Kan^r^	This study
pEC1	pUC18 derivative, source of the *ermB* gene, Ap^r^	Bruckner
pBT2	Shuttle vector, temperature sensitive, Ap^r^ Cm^r^	Bruckner
pBTairSR	pBT2 containing upstream and downstream fragments of *airSR* and *ermB* gene, for *airSR* mutagenesis, Ap^r^ Cm^r^ Em^r^	This study
pLI50	Shuttle cloning vector, Ap^r^ Cm^r^	Addgene
pLIairSR	pLI50 with *airSR* ORF and its promoter, Ap^r^ Cm^r^	This study

^a^NARSA, Network on Antimicrobial Resistance in *Staphylococcus aureus*;

^b^Kan^r^, kanamycin-resistant; Ap^r^, ampicillin-resistant; Cm^r^, chloramphenicol-resistant; Em^r^, erythromycin-resistant.

For collecting cells from oxygen depletion conditions, anaerobic jar of 15 ml volume was used. Briefly, overnight cultures were diluted 1:100 into anaerobic jar containing 10 ml TSB. Resazurin was added to a final concentration of 0.0002% (w/v) as indicator for anaerobic conditions. The jars were incubated at 37°C with shaking. Initially, the cultures were in red color, and after about 6 hours incubation the red faded out completely, indicating that the oxygen was completely consumed. Then cells were collected after two more hours’ incubation.

### Construction of the *airSR* mutant and the complementary strain

Construction of the *airSR* mutant strain was performed as previously described [24]. Briefly, the upstream and downstream regions of *airSR* were amplified from *S. ureus* NCTC8325 genomic DNA, and linked with *ermB* to form an up-*ermB*-down fragment, which was subcloned into the shuttle vector pBT2 to generate pBTairSR. The plasmid was introduced by electroporation into *S. aureus* RN4220 for modification and subsequently introduced into *S. aureus* NCTC8325. The strains that had allelic replacement of *airSR* by *ermB* were screened as erythromycin-resistant and chloramphenicol-sensitive colonies, and were further verified by PCR and sequencing.

For the complementation of the *airSR* mutation, a 2265-bp fragment of the *airSR* gene containing the promoter region was amplified and cloned into pLI50 to generate pLIairSR, which was introduced into *S. aureus* RN4220 for modification and, subsequently, introduced into the *airSR* mutant strain. The primers used in this study are listed in Table 2.

**Table 2:** Primers used in this study

**Primer name**	**Oligonucleotide (5′-3′)**^**a**^	**Application**
up-airSR-f	CCGgaattcTACATCTTGTGCCTTAG	*airSR* deletion
up-airSR-r	ATTTGAGatcgatAATGTTCAG	*airSR* deletion
down-airSR-f	CGATTTAAGTggtaccGTTGCATGATGTG	*airSR* deletion
down-airSR-r	CGCggatccCCTTAAGTTGTTGGAA	*airSR* deletion
Em-f	CGGatcgatGATACAAATTCCCCGTAGGC	*airSR* deletion
Em-r	CGGggtaccGAAATAGATTTAAAAATTTCGC	*airSR* deletion
c-airSR-f	CGCggatccATCGTCGCCAGTATG	ΔairS complementation
c-airSR-r	CCGgaattcTGAAGCGAAAGTAAATG	ΔairS complementation
e-airR-f	GGAATTCcatatgAACAAAGTAATATT	expression of AirR
e-airR-r	CCGctcgagAATCAACTTATTTTCCA	expression of AirR
e-airS-f	GGGAATTCcatatgATGGAACAAAGGACGCGACTAG	expression of AirS
e-airS-r	CCGctcgagCTATTTTATAGGAATTGTGAATTG	expression of AirS
RTQ-cap5B-f	GCTTATTGGTTACTTCTGA	real-time RT PCR
RTQ-cap5B-r	GTTGGCTTACGCATATC	real-time RT PCR
RTQ-cap5D-f	ATATGCCAGTGTGAGTGA	real-time RT PCR
RTQ-cap5D-r	CGGTCTATTGCCTGTAAC	real-time RT PCR
RTQ-lytM-f	CATTCGTAGATGCTCAAGGA	real-time RT PCR
RTQ-lytM-r	CTCGCTGTGTAGTCATTGT	real-time RT PCR
RTQ-640-f	TGATGGGACAGGAGT	real-time RT PCR
RTQ-640-r	TATTGTGCCGCTTCT	real-time RT PCR
RTQ-953-f	GTCATTGAGCACGATTTATT	real-time RT PCR
RTQ-953-r	TCTGGGCGGCTGTAA	real-time RT PCR
RTQ-pbp1-f	AGTCAGCGACCAACATT	real-time RT PCR
RTQ-pbp1-r	AAGCACCTTCTTGAATAGC	real-time RT PCR
RTQ-murD-f	TTCAGGAATAGAGCATAGA	real-time RT PCR
RTQ-murD-r	AACCACCACATAACCAA	real-time RT PCR
RTQ-1148-f	GCCGAAGTGACATAC	real-time RT PCR
RTQ-1148-r	AAGCACCGACTGATA	real-time RT PCR
RTQ-ddl-f	TAGGGTCAAGTGTAGGT	real-time RT PCR
RTQ-ddl-r	GTCGCTTCAGGATAG	real-time RT PCR
RTQ-pta-f	AAAGCGCCAGGTGCTAAATTAC	real-time RT PCR
RTQ-pta-r	CTGGACCAACTGCATCATATCC	real-time RT PCR
p-cap5A-f	TCATCTAACTCACCTGAAATTACAAAA	EMSA
p-cap5A-r	TTTCCATTATTTACCTCCCTTAAAAA	EMSA
p-ddl-f	CAAACTCCTTTTATACTC	EMSA
p-ddl-r	GTCATTTCGTTTTCCT	EMSA
p-pbp1-f	GATTCAATAGAACAAGCGATT	EMSA
p-pbp1-r	AGCTACACGTAATTTCGCGCTT	EMSA
p-lytM-f	GAATCGCGAACATGGACGAA	EMSA
p-lytM-r	GCAATCGCTGCTGCTGTTAA	EMSA

^a^The sequences in lowercase letters refer to the restriction endonuclease recognition sites.

### Triton X-100-induced autolysis assay

Triton X-100-stimulated autolysis was measured as described previously [25] with modifications. The cells (four replicates) were grown in TSB to the early exponential (OD_600_ = 1.0) phase at 37°C with constant shaking (220 rpm). The cells were collected by centrifugation, washed twice in 0.05 M Tris–HCl buffer (pH 7.5), resuspended in an equal volume of Tris–HCl buffer (0.05 M, pH 7.5) containing 0.05% (w/v) Triton X-100 (Sigma-Aldrich, St. Louis, MO, USA), and incubated at 37°C with constant shaking (220 rpm). The decrease in the optical density at 600 nm (OD_600_) was measured each hour using a microplate reader (Elx800, Bio-Tek, Winooski, VT, USA). The experiment was repeated at least three times with similar results.

### Vancomycin susceptibility assay

For the growth experiments, overnight cultures of *S. aureus* were diluted to 1.0 × 10^7^ colony-forming units (CFU)/ml in Mueller-Hinton (MH) broth medium (BD) with or without vancomycin, and inoculated into 50 ml flasks in a final volume of 10 ml. The flasks were incubated at 37°C with constant shaking (220 rpm). The growth was monitored each hour by measuring the OD_600_ using a spectrophotometer (DU 730, Beckman Coulter, Brea, CA, USA). For the plate sensitivity assays, overnight cultures were collected by centrifugation and adjusted to 1.0 × 10^7^ CFU/ml with MH. Each culture followed 4 tenfold serial dilutions, and 1 μl of each sample was spotted onto a MH agar plate that contained 0 or 0.6 μg/ml of vancomycin. All the plates and cultures were incubated at 37°C for 24 hours before the colonies were counted. These assays were repeated at least three times with similar results.

### Total RNA isolation, real-time RT PCR, and microarray processing

For the total RNA isolation, the overnight cultures of *S. aureus* were diluted 1:100 in TSB and then grown to the exponential phase until collected. The cells were processed with 1 ml TRIzol (TaKaRa, Kyoto, Japan) in combination with 0.1-mm-diameter-silica beads in a FastPrep-24 Automated system (MP Biomedicals Solon, OH, USA), and residual DNA was removed with RNase free DNaseI (TaKaRa, Kyoto, Japan). For the reverse transcription, the cDNAs were synthesized using a PrimeScript 1st Strand cDNA Synthesis Kit (TaKaRa). The real-time PCR was performed with SYBR Premix Ex Taq (TaKaRa) using the StepOne Real-Time PCR System (Applied Biosystems, Carlsbad, CA, USA). The quantity of cDNA measured using real-time PCR was normalized to the abundance of *pta* cDNA [26]. The real-time PCR assays were repeated at least three times. The microarray processing and data analysis were conducted by the Biochip Company of Shanghai, China. The microarray data was uploaded to Gene Expression Omnibus (GEO) with accession number: GSE51197.

### Purification of AirR and AirS

6-His-tagged AirR was cloned and purified using standard procedures. The full-length *airR* ORF was amplified by PCR with the e-airR-f and e-airR-r primers from *S. aureus* NCTC8325 genomic DNA, cloned into the expression vector pET28a (+) (Novagen, Merck, Darmstadt, Germany), and transformed into *E. coli* BL21 (DE3). The transformant was grown in LB at 37°C to an OD_600_ of 0.4 and induced with 0.5 mM isopropyl-β-D-1-thiogalactopyranoside (IPTG) at 37°C for an additional three hours. The cells were harvested and lysed by sonication in a lysis buffer (20 mM Tris–HCl, pH 8.0, 200 mM NaCl). The 6-His-tagged AirR protein was purified with a nickel-nitrilotriacetic acid agarose solution (Qiagen, Valencia, CA, USA) following the manufacturer’s recommendation. The bound protein was eluted with an elution buffer (200 mM imidazole, 20 mM Tris–HCl, pH 8.0, 200 mM NaCl). The imidazole in the eluent was removed using a Centrifuge Biomax-5 column (Millipore, Billerica, MA, USA), and the AirR protein solution was supplemented with 30% glycerol and stored at −80°C until use.

The full-length *airS* ORF was amplified using PCR with the e-airS-f and e-airS-r primers from *S. aureus* NCTC8325 genomic DNA, cloned into the expression vector pET28a (+), and transformed into *E. coli* BL21 (DE3). Purification of 6-His-tagged AirS was performed following the procedures of AirR purification except an overnight induction of 0.5 mM IPTG at 16°C. The purity of the proteins was determined by SDS-PAGE, and the protein concentration was determined using the BCA assay with bovine serum albumin as the standard.

### AirR phosphorylation *in vitro*

For AirR phosphorylation *in vitro*, we used lithium potassium acetyl phosphate as phosphoryl group donor. Briefly, 10 μM AirR was equilibrated in buffer containing 50 mM Tris at pH 7.4, 50 mM KCl, 5 mM MgCl_2_, and 10% glycerol (phosphorylation buffer). Lithium potassium acetyl phosphate (Sigma-Aldrich, St. Louis, MO, USA) was added to a final concentration of 50 mM, and this mixture was incubated for 60 min at 37°C [27].

We also used AirS for AirR phosphorylation *in vitro*. Briefly, 10 μl phosphorylation buffer containing 2 μM AirS and 10 mM ATP was used to initiate the autophosphorylation of AirS. After incubation at 25°C for 5 min, 10 μM AirR was added and the incubation was continued for another 10 min [22].

### Electrophoretic mobility shift assay

The DNA fragments containing the promoter region were amplified from the *S. aureus* NCTC8325 genomic DNA. The PCR products were labeled using a digoxigenin (DIG) gel shift kit (Roche, Indianapolis, IN, USA) according to the manufacturer’s instructions. The labeled fragment was incubated at 25°C for 15 min with various amounts of AirR in 10 μl of incubation buffer (10 mM Tris–HCl, pH 8.0, 100 mM NaCl, 1 mM EDTA). After incubation, the mixtures were electrophoresed in a 5% native polyacrylamide gel in 0.5 × Tris-borate-EDTA (TBE) buffer. The band shifts were detected and analyzed according to the manufacturer’s instructions. The images were obtained using ImageQuant LAS 4000 mini (GE, Piscataway, NJ, USA). The unlabeled fragments of each promoter were added to the labeled fragments at a ratio of approximately 50:1, respectively, as specific competitors (SCs). The unlabeled fragments of the *pta* ORF region (50-fold) were added as non-specific competitors (NCs).

### Statistics

The data were analyzed using the *T*-test analysis of variance, with a *P* value of < 0.05 considered significant (one asterisk), *P* < 0.01 (two asterisks).

## Results

### Transcriptional profile of the *airSR* mutated strain

To investigate the function of AirSR, we performed a cDNA microarray analysis using total RNA from the exponential growth stage. The microarray results indicated that approximately 190 genes were up-regulated (ratio > 2.0) and 290 genes were down-regulated (ratio < −2.0). We used Clusters of Orthologous Groups of proteins (COGs) of NCBI for protein function prediction. The expression levels of 29 cell wall metabolism-related genes were altered in the *airSR* mutant. The majority of these genes were down-regulated, including members of the capsular polysaccharide synthesis operon (*cap* operon), penicillin-binding protein 1 (*pbp1*), and other enzymes that are responsible for the biosynthesis of murein sacculus and peptidoglycan. The detailed results are listed in Table 3. These data suggest that *airSR* plays an important role in cell wall biosynthesis.

**Table 3:** **Cell wall synthesis-related genes that were differentially expressed in the *airSR *** **mutant compared to the NCTC8325 wild-type**

**Gene**		**Product**	**ΔairSR/WT ratio**^**a**^
SAOUHSC_00114	*cap5A*	Capsular polysaccharide biosynthesis protein, putative	−3.61
SAOUHSC_00115	*cap5B*	Capsular polysaccharide synthesis enzyme Cap5B	−2.86
SAOUHSC_00116	*cap8C*	Capsular polysaccharide synthesis enzyme Cap8C	−2.91
SAOUHSC_00117	*cap5D*	Capsular polysaccharide biosynthesis protein Cap5D	−2.4
SAOUHSC_00119	*cap8F*	Capsular polysaccharide synthesis enzyme Cap8F	−2.34
SAOUHSC_00122	*cap5I*	Capsular polysaccharide biosynthesis protein Cap5I	−2.1
SAOUHSC_00124	*cap5K*	Capsular polysaccharide biosynthesis protein Cap5K	−2.18
SAOUHSC_00126	*cap8M*	Capsular polysaccharide biosynthesis protein Cap8M	−2.02
SAOUHSC_00127	*cap5N*	Cap5N protein/UDP-glucose 4-epimerase, putative	−2.14
SAOUHSC_00222	*tagB*	TagB protein, putative	2.24
SAOUHSC_00295	*nanA*	N-acetylneuraminate lyase	−2.02
SAOUHSC_00469	*spoVG*	Regulatory protein SpoVG	−2.51
SAOUHSC_00545	*sdrD*	sdrD protein, putative	−3.68
SAOUHSC_00640	*tagA*	Teichoic acid biosynthesis protein	−2.08
SAOUHSC_00812	*clfA*	Clumping factor, ClfA	−4.72
SAOUHSC_00918		Truncated MHC class II analog protein	2.15
SAOUHSC_00953		Diacylglycerol glucosyltransferase	−2.21
SAOUHSC_00974		Glycosyl transferase, group 1	4.24
SAOUHSC_01106	*murI*	Glutamate racemase, MurI	−2.12
SAOUHSC_01145	*pbp1*	Penicillin-binding protein 1	−2.05
SAOUHSC_01147	*murD*	UDP-N-acetylmuramoylalanine--D-glutamate ligase, MurD	−2.58
SAOUHSC_01148	*ftsQ*	Cell division protein, putative	−2.38
SAOUHSC_01346		Glycine betaine transporter, putative	4.62
SAOUHSC_01400		Alanine racemase, putative	−2.81
SAOUHSC_02317	*murF*	UDP-N-acetylmuramoylalanyl-D-glutamyl-2,6-diaminopimelate--D-alanyl-D-alanyl ligase	−2.3
SAOUHSC_02318	*ddl*	D-alanyl-alanine synthetase A	−2.34
SAOUHSC_02399	*glmS*	Glucosamine--fructose-6-phosphate aminotransferase	−2.05
SAOUHSC_02444		Osmoprotectant transporter, BCCT family, opuD-like protein	−2.86
SAOUHSC_02998	*cap5C*	Capsular polysaccharide biosynthesis protein, Cap5C	2.04

^a^ “-” indicates down-regulated in the *airSR* mutant.

### Autolysis rate induced by Triton X-100

**Figure 1:**
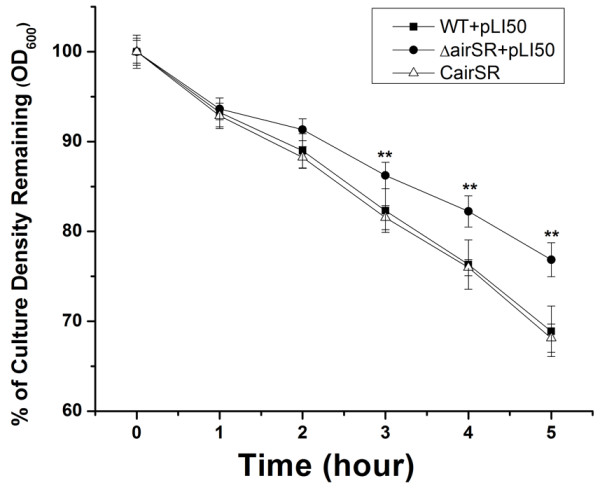
**The Triton X-100 induced autolysis.** The wild-type, the *airSR* mutant, and the complementary strain in Tris–HCl buffer containing 0.05% Trition X-100 at 37°C. (**indicates *P* < 0.01).

To test whether cell wall biosynthesis was affected, we examined Triton X-100-induced autolytic activity. The *airSR* mutant exhibited decreased autolysis rates compared with the wild-type strain. This phenomenon was restored by introducing a plasmid that contained the *airSR* operon (Figure 1), suggesting that cell wall turnover was affected by the inactivation of *airSR*.

### Viability of the *airSR* mutant in the presence of vancomycin

**Figure 2:**
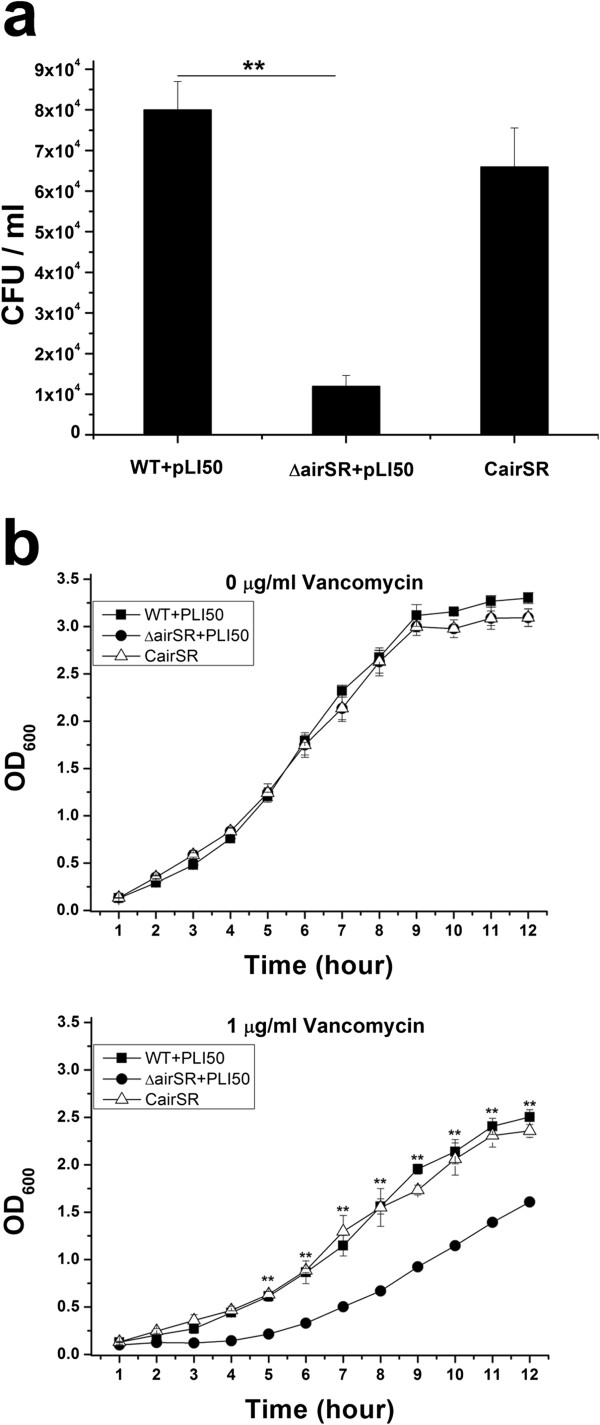
**Vancomycin susceptibility assay. (a)** Colony counts (CFU/ml) of WT, the *airSR* mutant, and the *airSR* complementary strains on MH agar plates containing vancomycin (0.6 μg/ml). The colonies were counted after incubation at 37°C for 24 hours. **(b)** The growth of the wild-type, the *airSR* mutant*,* and the *airSR* complementary strains in MH broth at 37°C. Vancomycin concentrations of 0 or 1.0 μg/ml. (**indicates *P* < 0.01).

Since vancomycin is an important antibiotic that targets *S. aureus* cell wall, we tested the viability of *S. aureus* in MH agar plates with vancomycin. The wild-type and the *airSR* mutant were able to grow at a maximum concentration of 0.6 μg/ml vancomycin, whereas the *airSR* mutant formed significantly fewer colonies (Figure 2a). We also tested cell growth in MH broth containing various concentrations of vancomycin. The cells were incubated in MH broth at an inoculum of 1 × 10^7^ CFU/ml, with constant shaking at 37°C. No significant difference was observed when cells grew in MH broth without vancomycin. The *airSR* mutant exhibited a clear growth defect compared to the wild-type in the medium containing 1.0 μg/ml vancomycin (Figure 2b). Taken together, these results indicate that the *airSR* inactivation reduced the ability of the bacteria to survive in the presence of vancomycin.

### Transcriptional analysis using real-time RT PCR

**Figure 3:**
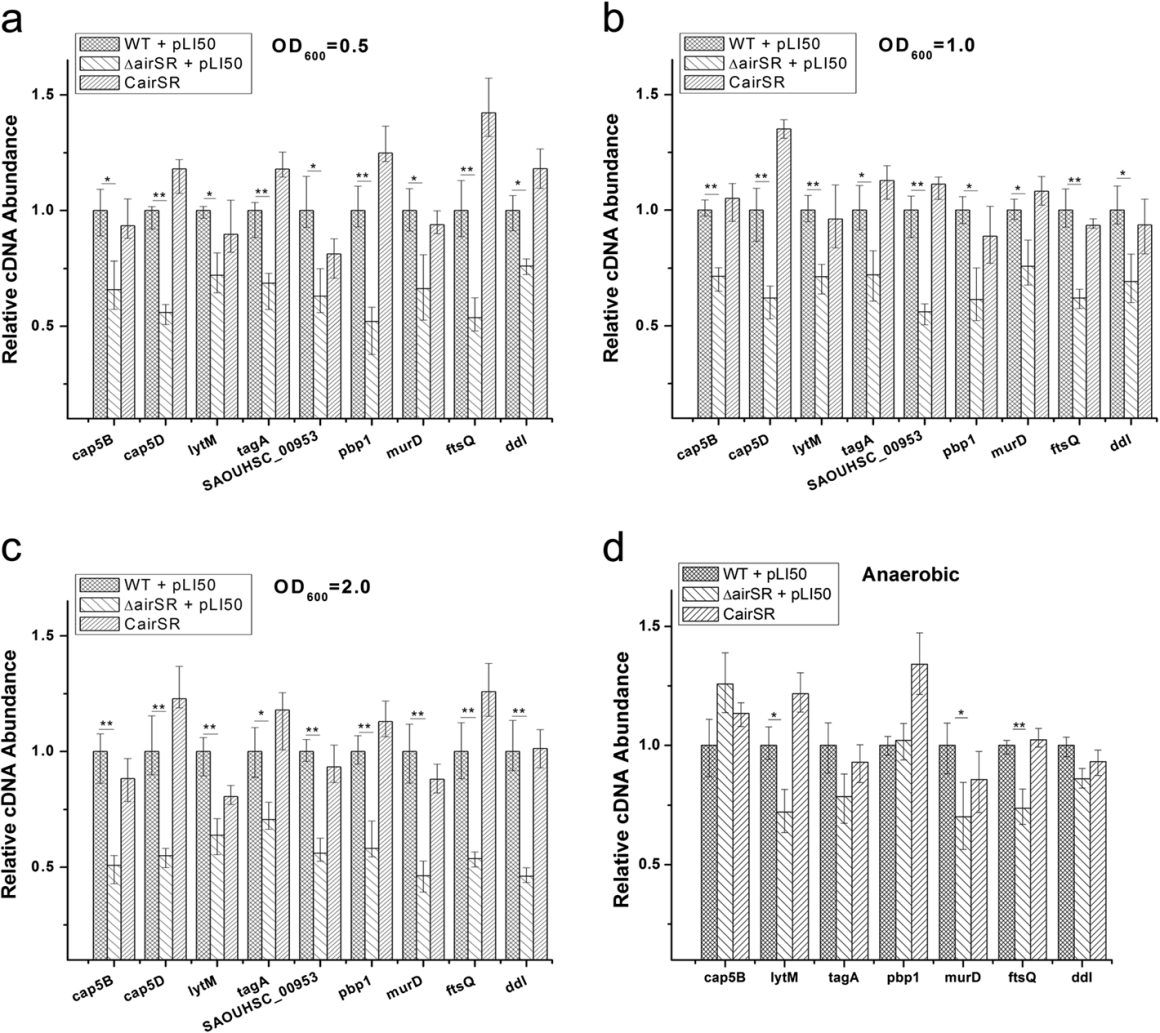
**Transcriptional level of several cell wall-related genes.** Comparison of the relative transcription levels of several cell wall biosynthesis- and hydrolysis-related genes in the wild-type, the *airSR* mutant, and the *airSR* complementary strains. **(a)**, **(b)**, and **(c)** transcriptional levels under aerobic conditions in different time courses; **(d)** transcriptional levels under anaerobic conditions. (*indicates P < 0.05; **indicates P < 0.01).

To verify the microarray results, mRNA levels from different growth stages were examined using real-time RT PCR. The mRNA levels of certain cell wall-related genes, including *cap5B*, *cap5D*, *tagA*, SAOUHSC_00953, *pbp1*, *murD*, *ftsQ*, and *ddl*, were significantly reduced (Figure 3a, b,c). These results were in accordance with the microarray results. We also investigated the transcriptional levels of various peptidoglycan hydrolase-coding genes. Only *lytM* was down-regulated, as indicated by real-time PCR (Figure 3a,b,c), while *atl sle1* and *lytN* showed no obvious changes in expression (data not shown).

When we used cells collected from oxygen depletion conditions for real-time RT PCR, we found that only three genes (*lytM*, *murD*, *ftsQ*) showed the same down-regulation as under aerobic conditions (Figure 3d). This may suggest that AirSR needs oxygen to exert its regulation ability.

We also compared the transcriptional level of several genes from the real-time RT PCR result and the microarray data, and found a positive correlation between the two techniques (Additional file 1).

### The binding of AirR to the target genes

**Figure 4:**
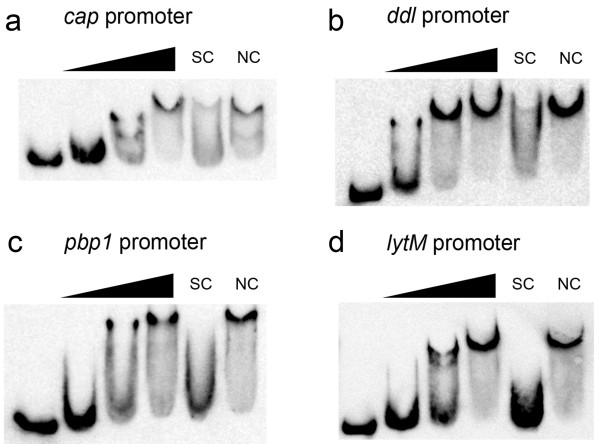
**Electrophoretic mobility shift assay for AirR.** The first lane was the free DNA probe (2 nM); the second to fourth lanes were the DNA probe with increasing amounts of AirR (0.3, 0.6, and 1.2 μM); the fifth lane was the same as the fourth lane but with the addition of a 50-fold excess of unlabeled probes as specific competitors (SCs). The sixth lane was as the same as the fourth lane but with the addition of a 50-fold excess of unlabeled *pta* ORF region fragments as non-specific competitor. (NC). **(a)** EMSA with *cap* promoter; **(b) ***ddl* promoter; **(c) ***pbp1* promoter; **(d) ***lytM* promoter.

We cloned and purified a His-tagged AirR to perform gel shift assays. DNA probes containing the putative promoters of several target genes were amplified. A clearly shifted band of DNA was visible after incubation of AirR with DNA probes containing the *cap* promoter (Figure 4a). The intensity of the shifted band increased as the amount of AirR was higher. This shifted band disappeared in the presence of an approximately 50-fold excess of unlabeled *cap* promoter DNA but not in the presence of 50-fold excess of an unlabeled coding sequence DNA of *pta*. These data suggest that AirR can specifically bind to the *cap* promoter region.

Similar assays were performed using DNA fragments of the promoter region of *ddl* and *pbp1*, two other genes that encode cell wall biosynthesis-related proteins. Similar promoter DNA band shift patterns were observed with the *ddl* and *pbp1* promoters (Figure 4b,c), suggesting that AirR can bind to these promoters. The promoter region of *lytM* was amplified and used as a gel shift probe. The result indicated that AirR can specifically bind to the *lytM* promoter (Figure 4d).

To test the effect of phosphorylation of AirR, same amount of AirR or AirR-P obtained from both lithium potassium acetyl phosphate and AirS were used for EMSA of *cap* promoter. The shift band from different proteins did not show obvious difference (Additional file 2), which is consistent with the observation by another group [23].

## Discussion

Our study shows a direct connection between cell wall metabolism and AirSR. More than 20 genes that are related to cell wall metabolism were down-regulated in the airSR mutant, as shown by microarray analysis. Real-time RT PCR experiments confirmed the transcript level changes of several genes (*cap5B*, *cap5D*, *tagA*, SAOUHSC_00953, *pbp1*, *murD*, *ftsQ*, and *ddl*). Real-time RT PCR indicated that the transcription of a major autolysin, LytM, was down-regulated in the *airSR* mutant. This result is consistent with the observation of a decreased autolysis rates induced by Triton X-100 in the *airSR* mutant. The gel-shift assays indicated that AirR can directly bind to the promoter regions of *cap*, *ddl*, *pbp1*, and *lytM*. These results suggest that AirSR enhances cell wall synthesis and degradation.

We performed the phylogenetic footprinting using promoter sequences from orthologous target genes in *Staphylococci*. Analysis of these sequences using CLUSTAL Multiple Sequence alignment and MEME [28] suggests that a motif “AAATNNAAAATNNNNTT” may represent the binding sequence of AirR (see Additional file 3). In our further study, we will use footprinting to identify the exact binding sequence and motif and then search genome wide for more potential targets.

Cell wall synthesis is crucial for bacterial division and growth, and it is a very important target of antibiotics, such as penicillin, vancomycin, and teicoplanin. With the increase in the number of MRSA strains, vancomycin has become the first choice to treat staphylococcal infections. The use of vancomycin has led to the emergence of vancomycin-intermediate *Staphylococcus aureus* (VISA). Typically, VISA exhibits thick cell walls and reduced autolysis rates. Our study demonstrated that the *airSR* mutation exhibited both reduced viability in vancomycin and attenuated autolysis. We speculated that, the affected expression of cell wall metabolism-related genes owing to the *airSR* mutation caused the reduction in cell viability due to vancomycin. Attenuated autolysis may be a compensatory mechanism for the affected cell wall synthesis. The reduction of viability in the presence of vancomycin and the attenuation of autolysis are two independent outcomes of the *airSR* mutation.

One other research group previously designated *airSR* as *yhcSR* and reported that it was an essential TCS [20]. However, there are reports of an *airSR* mutation in several strains including Newman [22], MW2 [29], a clinically isolated strain 15981 [9], and NCTC8325, indicating that *AirSR* is unlikely to be essential in all strain backgrounds. Early research on *airSR* reported that this TCS is involved in the regulation of the nitrate respiratory pathway [21] or in the direct regulation of the *lac* and *opuCABCD* operons [23]. Our microarray results indicated the down-regulation of the *nar* and *nre* operons in the *airSR* mutant, which is consistent with the report that *airSR* can positively regulate the nitrate respiratory pathway [21]. Our microarray data, however, did not show that *airSR* can regulate *lac* or *opuC* operons (data not shown). Another group that first named this TCS *airSR* described *airSR* as an oxygen sensing and redox-signaling regulator. Though they stated that *airS* contains a Fe-S-cluster essential for oxygen sensing and is only active in the presence of oxygen *in vitro*, they found that the *airR* mutant only affects gene expression under anaerobic conditions in strain Newman [22]. In contrast, our results showed that the expression of cell wall metabolism-related genes was not changed under anaerobic conditions (Figure 3d), but only under aerobic conditions (Figure 3a,b,c). After further comparison of their microarray data with ours, we found that, *cap* operon, *pbp1*, and *lytM* were under negative control of AirR in their strain but positive control in our strain; *saeSR*, *agr*, and *RNAIII* were under negative control in their strain but not changed in our strain; *spa* and *hlgC* are under positive control of AirR in both strains (see Additional file 4). The discrepancy may suggest that the regulatory activity of AirR is strain specific. Why AirSR acts differently in different strains is still not clear. Our speculation is that inactivation of sigma B in NCTC8325 may contribute to the different activity of AirSR in NCTC8325 and Newman. But this speculation needs further study.

WalKR is an important TCS that positively controls cell wall biosynthesis and turnover, including directly controlling lytM [12]. Alterations in the expression of WalKR as well as WalKR mutations at amino acid sequence can lead to a change in susceptibility to vancomycin [19,30]. AirSR and WalKR exhibit similar functions. Our microarray data indicate that the WalKR expression level is unchanged in the *airSR* mutant, and there is no report so far that WalKR regulates AirSR, suggesting that the two TCSs may regulate cell wall biosynthesis independently.

## Conclusions

The *airSR* mutant exhibited reduced autolysis and down-regulation in many cell wall metabolism-related genes in *S. aureus* NCTC8325. And AirR can directly bind to the promoter region of some of these cell wall metabolism genes. These findings demonstrate that AirSR is a positive regulator in cell wall biosynthesis and turnover in *S. aureus* NCTC8325. The *airSR* mutant exhibited reduced viability in the presence of vancomycin, suggesting that AirSR could be a new target for controlling *S. aureus* infection.

## Competing interests

The authors declare that they have no competing interests.

## Authors’ contributions

HS, TX, and BS designed the study. HS and YY performed laboratory work. HS, YY, and TX performed data analysis. HS and YY wrote the manuscript. TX and BS critically revised the manuscript. All authors read and approved the final manuscript.

## Supplementary Material

Additional file 1 **Correlationship between microarray data and the real-time RT PCR result.** The transcriptional level of 11 genes from both microarray and real-time RT PCR were log2 transformed and plotted against each other. A linear fit analysis was performed to check the correlation between the two methods. R^2^ = 0.9678. Click here for file

Additional file 2 **EMSA of *cap *promoter with unphosphorylated and phosphorylated AirR.** The first lane was the free DNA probe (2 nM); the second to fourth lanes were the DNA probe with increasing amounts of unphosphorylated AirR (0.25, 0.5, and 1 μM); the fifth to seventh lanes were the DNA probe with increasing amounts of lithium potassium acetyl phosphate phosphorylated AirR (0.25, 0.5, and 1 μM); the eighth to tenth lanes were the DNA probe with increasing amounts of AirS phosphorylated AirR (0.25, 0.5, and 1 μM). Click here for file

Additional file 3 **Phylogenetic footprinting of AirR binding sequences.** The sequences of orthologous target genes were analyzed by CLUSTAL Multiple Sequence alignment and MEME. Potential binding sequence of AirR was listed below. Click here for file

Additional file 4 **Comparison of microarray result of previous report.** The table contains both microarray data and the verification result of real-time RT PCR. Click here for file
