# Supplementary figures and images for: Modulation of cell wall synthesis and susceptibility to vancomycin by the two-component system AirSR in Staphylococcus aureus NCTC8325
Source: BMC Microbiol. 2013 Dec 10;13:286. doi: 10.1186/1471-2180-13-286 (PMC4029521; doi:10.1186/1471-2180-13-286)

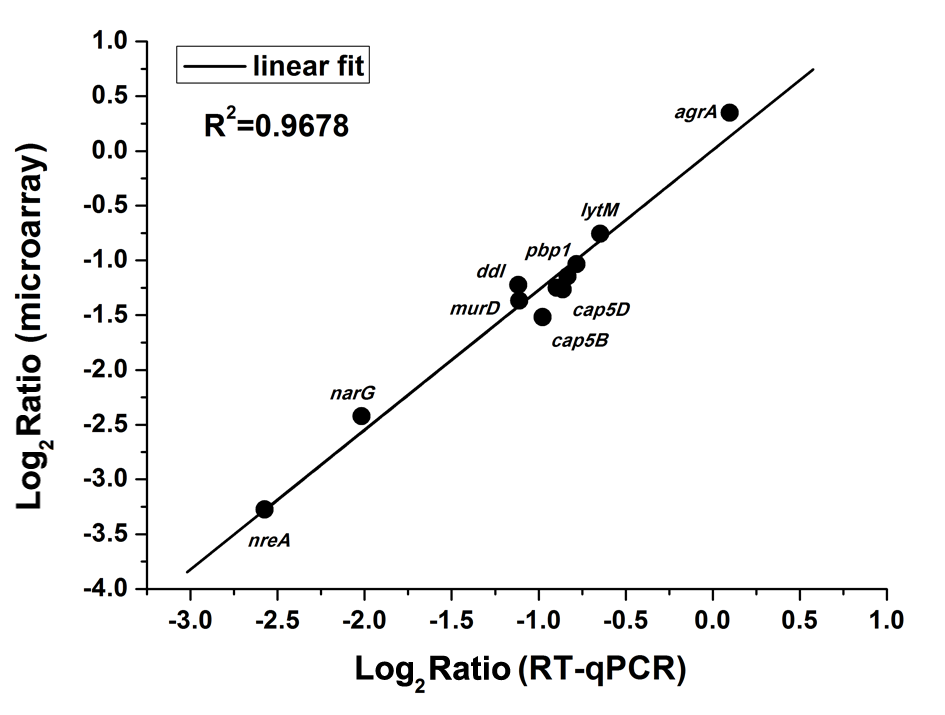

Supplement: Additional file 1 — Correlationship between microarray data and the real-time RT PCR result. The transcriptional level of 11 genes from both microarray and real-time RT PCR were log2 transformed and plotted against each other. A linear fit analysis was performed to check the correlation between the two methods. R2 = 0.9678. [file 1471-2180-13-286-S1.tiff]

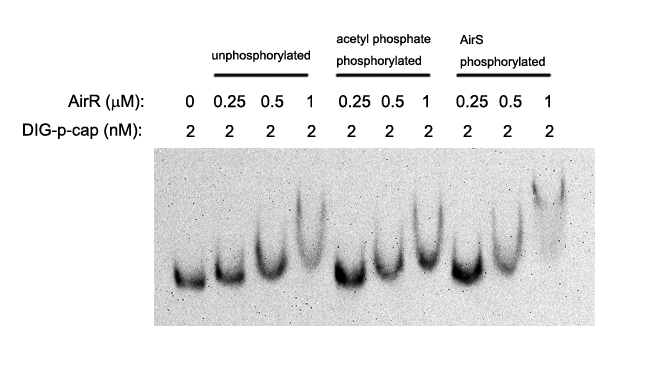

Supplement: Additional file 2 — EMSA of cap promoter with unphosphorylated and phosphorylated AirR. The first lane was the free DNA probe (2 nM); the second to fourth lanes were the DNA probe with increasing amounts of unphosphorylated AirR (0.25, 0.5, and 1 μM); the fifth to seventh lanes were the DNA probe with increasing amounts of lithium potassium acetyl phosphate phosphorylated AirR (0.25, 0.5, and 1 μM); the eighth to tenth lanes were the DNA probe with increasing amounts of AirS phosphorylated AirR (0.25, 0.5, and 1 μM). [file 1471-2180-13-286-S2.tiff]
