# Supplementary material for: Modulation of cell wall synthesis and susceptibility to vancomycin by the two-component system AirSR in Staphylococcus aureus NCTC8325
Source: BMC Microbiol. 2013 Dec 10;13:286. doi: 10.1186/1471-2180-13-286 (PMC4029521; doi:10.1186/1471-2180-13-286)
Supplement: Additional file 3 — Phylogenetic footprinting of AirR binding sequences. The sequences of orthologous target genes were analyzed by CLUSTAL Multiple Sequence alignment and MEME. Potential binding sequence of AirR was listed below. [file 1471-2180-13-286-S3.pdf]

Conservative region of *ddl* promoter in *Staphylococci*

```
                                                                     Section 1
                    (1) 1        10        20        30        40        50
p-ddl-8325          (1) --------------CTCACTAATGTTTATATCA--ATTTTACATGACTTT
p-ddl-Mu50          (1) --------------CTCACTAATGTTTATATCA--ATTTTACATGACTTT
p-ddl-MW2           (1) --------------CTCACTAATGTTTATATCA--ATTTTACATGACTTT
p-ddl-S.capitis     (1) -----------------ATAAATAG-TCTACATTGATTTTACATGACTTT
p-ddl-S.caprae      (1) -----------------ATAAATAG-TCTACATTGATTTTACATGACTTT
p-ddl-S.epidermidis (1) ---TTCATTCATATTTTCTAATCAAATATATTAATTTTACATGACTTC
p-ddl-S.pasteuri    (1) ATAATCTTTCATCTTTCATCCATAATTATATAACGATTTTACATGACTTT
p-ddl-S.warneri     (1) -TAATCATTCATCTTTCATCCATAATTATATAACGATTTTACATGACTTT
p-ddl-S.simiae      (1) -------------CCACACTTATAGTTATTTTA--ATTTTACATGACTTT
p-ddl-N315          (1) --------------CTCACTAATGTTTATATCA--ATTTTACATGACTTT
                                                                     Section 2
                   (51) 51       60        70        80        90        100
p-ddl-8325         (35) TTAAAAATTAGCTAGAATATCACAGTGATATCAGCTATAGATTTCAATTT
p-ddl-Mu50         (35) TTAAAAATTAGCTAGAATATCACAGTGATATCAGCTATAGATTTCAATTT
p-ddl-MW2          (35) TTAAAAATTAGCTAGAATATCACAGTGATATCAGCTATAGATTTCAATTT
p-ddl-S.capitis    (33) TTTTAAATTTGCTAGAATATCAAAG---TGGCAAGTCGACAAATTATATA
p-ddl-S.caprae     (33) TTTTAAATTTGCTAGAATATCAAAG---TGGCAAGTCGACAAATTATATA
p-ddl-S.epidermidis (48) TTTTAAATTTGCTAGAATATCACAG---AATGATAGCACCTTATTAACTT
p-ddl-S.pasteuri   (51) ATAGAAATTTGCTAGAATATCAAGTCTAAATGCAATAACCAATTTATTTC
p-ddl-S.warneri    (50) ATAGAAATTTGCTAGAATATCAAGTTCAATTGCAATAACCATTTTATTTC
p-ddl-S.simiae     (36) TACTAAATTAGCTAGAATATCCAGATGATAAAACAACTAGAATACATAAC
p-ddl-N315         (35) TTAAAAATTAGCTAGAATATCACAGTGATATCAGCTATAGATTTCAATTT
                                                                     Section 3
                  (101) 101      110       120       130       140       150
p-ddl-8325         (85) GAATTAGGA---ATAAAATAGAAGGGAATA-TTGTTCTGATTATAAATGA
p-ddl-Mu50         (85) GAATTAGGA---ATAAAATAGAAGGGAATA-TTGTTCTGATTATAAATGA
p-ddl-MW2          (85) GAATTAGGA---ATAAAATAGAGGGGAATA-TTGTTCTGATTATAAATGA
p-ddl-S.capitis    (80) GAAATAAGCT--TTCTTTTCCATTCAAAG--GTAGAGGAATTAAAACAG-
p-ddl-S.caprae     (80) GAAATAAGCT--TTCTTTTCCATTCAAAG--GTAGAGGAATTAAAACAG-
p-ddl-S.epidermidis (95) TTAATATGTA--ATAGTCATAATAAAAATATGTATATTAGCGCAAATTG-
p-ddl-S.pasteuri  (101) ATAGAAAGT---ATTAGATTAAATA-TAGG-TTTGGTTAAACAACTTTG-
p-ddl-S.warneri   (100) TAAGGGAGT---ATTAGATTGAATAGTAGG-TTTGGTAAAAGAACTTTG-
p-ddl-S.simiae     (86) GAAATAACATTGATAATCTTAAAGAGAACT-GTAACATAATTAGATAGGG
p-ddl-N315         (85) GAATTAGGA---ATAAAATAGAAGGGAATA-TTGTTCTGATTATAAATGA
                                                                     Section 4
                  (151) 151      160       170       180       190       200
p-ddl-8325        (131) ATCAACATAGATACAGACACATAAGTCCTCGTTTTTAAAATGCAAAATAG
p-ddl-Mu50        (131) ATCAACATAGATACAGACACATAAGTCCTCGTTTTTAAAATGCAAAATAG
p-ddl-MW2         (131) ATCAACATAGATACAGACACATAACGCCTCGTTTTTAAAATGCAAAATAG
p-ddl-S.capitis   (125) --TCACTAAAAATTAGATTTATTG---ATGGATTAACAAATGCAATTGAT
p-ddl-S.caprae    (125) --TCACTAAAAATTAGATTTATTG---ATGGATTATCAAATGCAATTGAT
p-ddl-S.epidermidis (142) --TCATTAAAATTTAGTTCTATAT---TTAGATTATCAAATGCAATTGAA
p-ddl-S.pasteuri  (145) --CCATATATATTAATAGA-------------TTATAAAATGCAAAAGTC
p-ddl-S.warneri   (145) --CCCTTTATATTAATAGA-------------TTATAAAATGCAAAAGTC
p-ddl-S.simiae    (135) A-CGGTAAATATTCACATT-ATAGCGCTT--ATTTTGAAATGCAAAATAA
p-ddl-N315        (131) ATCAACATAGATACAGACACATAAGTCCTCGTTTTTAAAATGCAAAATAG
```

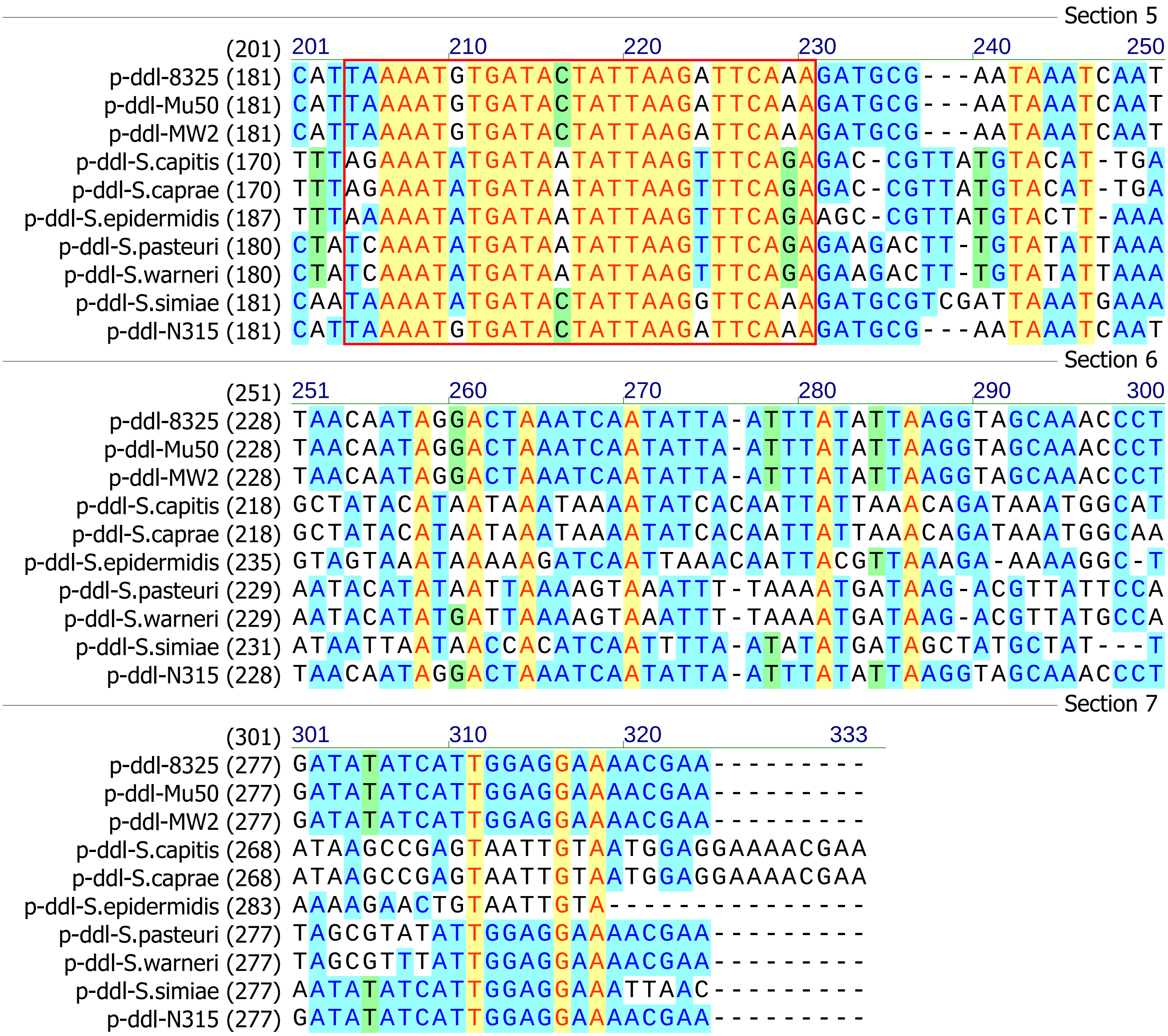

Potential AirR binding sequence

Among the conservative regions of *ddl* promoter squared in red, only the sequence under blue line can be found in other AirR direct targets. The sequences was analyzed using MEME and listed below. The sequence conservation, measured in bits, in shown as the height of a stack of letters at each base position.

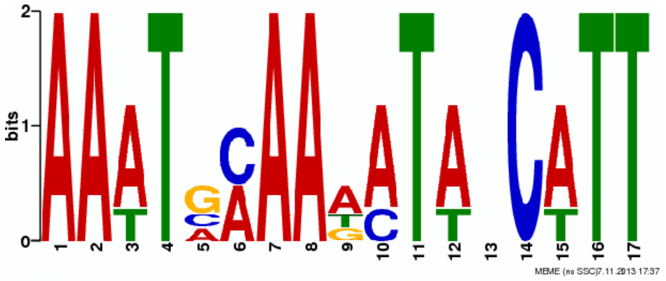

Name	p-value	Sites		
p-ddl	2.52e-09	GTTTTTA	AAATGCAAAATAGCATT	AAAATGTGAT
p-capA	1.77e-07	ATCAGTG	AAATCAAAGATATCATT	CAAACATCAT
p-pbp1	6.86e-07	ACTTTCG	AAATACAATATTCCATT	AGAAAAAGTG
p-lytM	1.11e-06	ACAGTTT	AATTGAAAACTAACTTT	AACTTTAATG
