# Supplementary material for: Modulation of cell wall synthesis and susceptibility to vancomycin by the two-component system AirSR in Staphylococcus aureus NCTC8325
Source: BMC Microbiol. 2013 Dec 10;13:286. doi: 10.1186/1471-2180-13-286 (PMC4029521; doi:10.1186/1471-2180-13-286)
Supplement: Additional file 4 — Comparison of microarray result of previous report. The table contains both microarray data and the verification result of real-time RT PCR. [file 1471-2180-13-286-S4.pdf]

	Previous report [b]				Our result		
	microarray ΔairR/Newman		RT-qPCR ΔairR/Newman		microarray ΔairSR/8325	RT-qPCR ΔairSR/8325	
	midlog	stationary	midlog	stationary		aerobic [c]	anaerobic
cap5A	4.92	45.71	2.81±0.53	5.76±0.08	-3.61	-1.97±0.07 [d]	1.26±0.13 [d]
RNAIII	3.57	3.39	16.99±0.09	10.99±0.07	-1.06	1.21±0.09	1.47±0.12
agrA	1.96				0.79	1.07±0.04	1.15±0.23
agrD		2.63			0.82		
saeS		3			0.93	0.89±0.11	1.05±0.07
saeR		2.27			0.93		
rsbW		2.77			-1.76		
rsbU [a]		1.87					
spa	-2.2	-3.96	-18±0.12	-9±0.08	-4.75	-2.57±0.17	-3.01±0.26
hlgC	-2.97	-2.08			-4.66	-2.94±0.33	-3.86±0.61
lytM	2.07				-1.69	-1.56±0.21	-1.39±0.15
pbp1		2.32			-2.05	-1.82±0.26	

[a] RsbU is inactive in 8325, so *rsbU* microarray result was not presented.

[b] Data is from "Sun F, Ji Q, Jones MB, Deng X, Liang H, Frank B, Telser J, Peterson SN, Bae T, He C: AirSR, a [2Fe-2S] cluster-containing two-component system, mediates global oxygen sensing and redox signaling in Staphylococcus aureus. Journal of the American Chemical Society 2012, 134(1):305-314."

[c] Data is obtained from total RNA of OD_{600} = 2.0

[d] It is RT-qPCR data of *cap5B*
